# Role of Bone Morphogenetic Protein 7 (BMP7) in the Modulation of Corneal Stromal and Epithelial Cell Functions

**DOI:** 10.3390/ijms19051415

**Published:** 2018-05-09

**Authors:** Bhavani S. Kowtharapu, Ruby Kala Prakasam, Radovan Murín, Dirk Koczan, Thomas Stahnke, Andreas Wree, Anselm G. M. Jünemann, Oliver Stachs

**Affiliations:** 1 Department of Ophthalmology, Rostock University Medical Center, 18057 Rostock, Germany; rubykala.prakasam@med.uni-rostock.de (R.K.P.); thomas.stahnke@med.unirostock.de (T.S.); anselm.juenemann@med.uni-rostock.de (A.G.M.J.); oliver.stachs@med.uni-rostock.de (O.S.); 2 Department of Medical Biochemistry, Jessenius Faculty of Medicine in Martin, Comenius University in Bratislava, 03601 Martin, Slovakia; murin@jfmed.uniba.sk; 3 Institute for Immunology, Rostock University Medical Center, 18057 Rostock, Germany; dirk.koczan@med.uni-rostock.de; 4 Institute for Anatomy, Rostock University Medical Center, 18057 Rostock, Germany; andreas.wree@med.uni-rostock.de

**Keywords:** corneal epithelial cells, corneal stromal fibroblasts, bone morphogenetic protein 7, gene expression profiling, wound healing, epithelial-to-mesenchymal transition

## Abstract

In the cornea, healing of the wounded avascular surface is an intricate process comprising the involvement of epithelial, stromal and neuronal cell interactions. These interactions result to the release of various growth factors that play prominent roles during corneal wound healing response. Bone morphogenetic proteins (BMPs) are unique multi-functional potent growth factors of the transforming growth factor-beta (TGF-β) superfamily. Treatment of corneal epithelial cells with substance P and nerve growth factor resulted to an increase in the expression of BMP7 mRNA. Since BMP7 is known to modulate the process of corneal wound healing, in this present study, we investigated the influence of exogenous rhBMP7 on human corneal epithelial cell and stromal cell (SFs) function. To obtain a high-fidelity expression profiling of activated biomarkers and pathways, transcriptome-wide gene-level expression profiling of epithelial cells in the presence of BMP7 was performed. Gene ontology analysis shows BMP7 stimulation activated TGF-β signaling and cell cycle pathways, whereas biological processes related to cell cycle, microtubule and intermediate filament cytoskeleton organization were significantly impacted in corneal epithelial cells. Scratch wound healing assay showed increased motility and migration of BMP7 treated epithelial cells. BMP7 stimulation studies show activation of MAPK cascade proteins in epithelial cells and SFs. Similarly, a difference in the expression of claudin, Zink finger E-box-binding homeobox 1 was observed along with phosphorylation levels of cofilin in epithelial cells. Stimulation of SFs with BMP7 activated them with increased expression of α-smooth muscle actin. In addition, an elevated phosphorylation of epidermal growth factor receptor following BMP7 stimulation was also observed both in corneal epithelial cells and SFs. Based on our transcriptome analysis data on epithelial cells and the results obtained in SFs, we conclude that BMP7 contributes to epithelial-to-mesenchymal transition-like responses and plays a role equivalent to TGF-β in the course of corneal wound healing.

## 1. Introduction

In the cornea, endogenously produced growth factors play a crucial role in modulating the functions of corneal epithelial and stromal cells [1,2,3]. A multitude of growth factors including epidermal growth factor (EGF), transforming growth factor-alpha and beta (TGF-α and -β), keratinocyte growth factor, fibroblast growth factor, hepatocyte growth factor, insulin-like growth factor, platelet-derived growth factor along with inflammatory cytokines such as interleukin (IL)-1, IL-6 and tumor necrosis factor (TNF)-α play important roles by influencing the migration, mitosis, proliferation and differentiation of corneal cells [4,5]. In conjunction, neurotrophic factors produced in the cornea elevate expression levels of vital growth factors and alter the corneal cell functions [6,7,8]. Similarly, corneal epithelial cell-derived growth factors also influence the function of corneal stromal cells [2].

TGF-β superfamily is one of the most important groups of cytokines involved in orchestrating the course of various essential cellular functions in the cornea [9,10]. The bone morphogenetic proteins (BMPs) are secreted, multifunctional, potent growth factors constituting the largest subgroup of the TGF-β superfamily [11]. The cellular responses stimulated by BMPs are mediated by two types of activated transmembrane serine/threonine kinase receptors, type I (BMPR-IA and BMPR-IB) and type II (BMPR-II) [12]. Subsequent signaling events are mediated by both, Smad-dependent and -independent pathways [13]. During BMP stimulated cellular signal transduction, the specifically triggered signaling pathway is reliant on the cellular activity, extracellular factors and crosstalk with other signaling cascades [14]. In general, BMP-mediated signaling is controlled intracellularly by methylation and inhibitory Smads and extracellularly by its antagonists [15,16]. BMPs are extensively expressed in the course of mammalian development and exert effect on numerous cellular functions with a wide range of biological activities on various cell types including development, morphogenesis, proliferation and extracellular matrix synthesis [17]. Throughout the development of the eye, BMP signaling has been shown to be pivotal in the regulation of early eye development [18], differentiation of retina [19], development of lens [20] and ciliary body [21]. Various BMPs (BMP2, -3, -4, -5 and -7) and their receptors have been shown to be expressed in the corneal epithelium and stroma [22,23]. BMPs, by inducing the expression of extracellular matrix components, also facilitate migration of cells during wound healing [24].

After injury, intracellular signaling cross-talk, due to the release of different growth factors at the wound site, collectively culminates to cell motility, migration and wound closure [24]. Likewise, in the course of growth factor-mediated signaling, cross-communication between different signaling pathways is eminent in different cell types which play an essential role in mediating activation of various signaling cascades and cellular functions [16,25]. For example, G-protein-coupled receptors and cAMP are known to use epidermal growth factor receptor (EGFR) to trigger downstream mitogenic signaling events [26,27,28]. In the same way, BMP7 also regulates the activation of EGFR [29]. In the cornea, EGFR transactivation through non-EGF ligands influences the differentiation of corneal stromal cells [30] as well as epithelial wound healing [31,32].

Of all the BMP family members, BMP7 functions as an indispensable signaling molecule during mammalian eye development [33]. Overexpression of BMP7 has been shown to effectively modulate cell proliferation [34], Smad signaling in mouse [35] and rabbit corneas [36]. Similar to TGF-β [37], BMP7 is also known to be involved in the progression of EMT [38] along with its role in wound healing [35,39]. Furthermore, in our previous study, we also observed differences in the expression of BMP7 during corneal epithelial and neuronal interactions [40]. Since BMP7 plays an important role as a trophic factor [41] as well as in sustaining the epithelial phenotype [42] and modulating the function of corneal cells, the present study is designed to investigate the influence of exogenous recombinant human BMP7 (rhBMP7) on corneal epithelial cells and corneal stromal fibroblasts (SFs) functions in vitro. Furthermore, we also performed transcriptome-wide gene-level expression profiling of corneal epithelial cells in the presence of rhBMP7 to obtain a high-fidelity expression profiling of activated biomarkers and pathways.

## 2. Results

### 2.1. Elevation of BMP7 mRNA Expression after Treatment of Telomerase-Immortalized Human Corneal Epithelial (hTCEpi) Cells with Substance P (SP) and Nerve Growth Factor (NGF)

**Figure 1:**
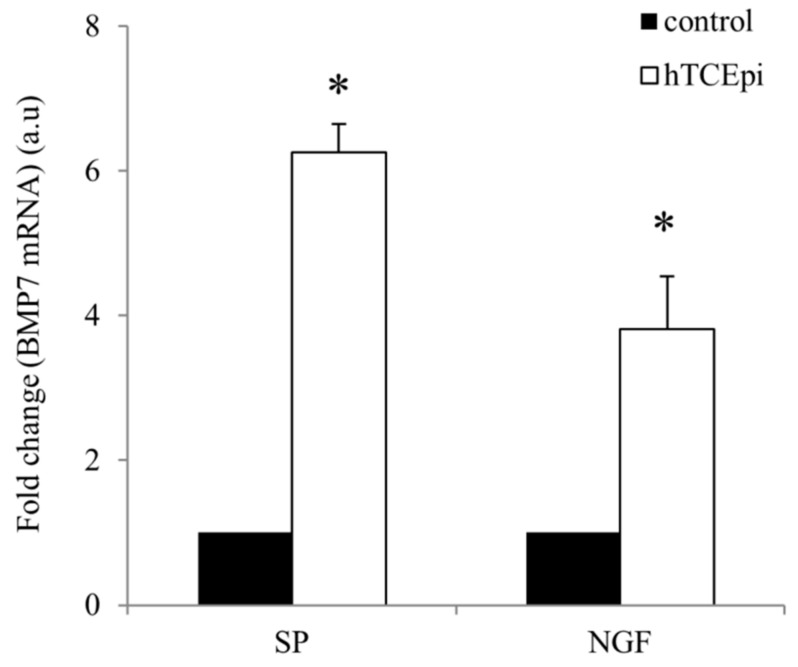
Relative expression levels of BMP7 mRNA in hTCEpi cells after treatment with SP (10^−5^ M) and NGF (100 ng/mL). Total RNA was isolated from SP and NGF treated hTCEpi cells after 24 h. Reverse transcription was performed using 1 µg of total RNA to prepare cDNA and qRT-PCR was performed to find differences in the expression levels of BMP7 mRNA. Relative expression pattern was analyzed by the comparative threshold cycle (2^−ΔΔ*C*t^) method. Expression values were represented as fold change over the control on an arbitrary scale after normalization with GAPDH. Data represent mean ± standard deviation of three independent experiments. The *p*-values of <0.05 were considered statistically significant and are indicated by asterisks (*).

In the presence of neuropeptides SP and NGF, hTCEpi cells showed enhanced expression of BMP7 mRNA. Corneal epithelial cells, after 24 h of treatment with SP, showed 6.2-fold increase in the expression of BMP7 mRNA whereas treatment with NGF also resulted to 3.8-fold increased expression compared to the control untreated cells (Figure 1).

### 2.2. Gene Expression Profiling (GEP) of Recombinant Human BMP7 (rhBMP7) Stimulated hTCEpi Cells 

**Figure 2:**
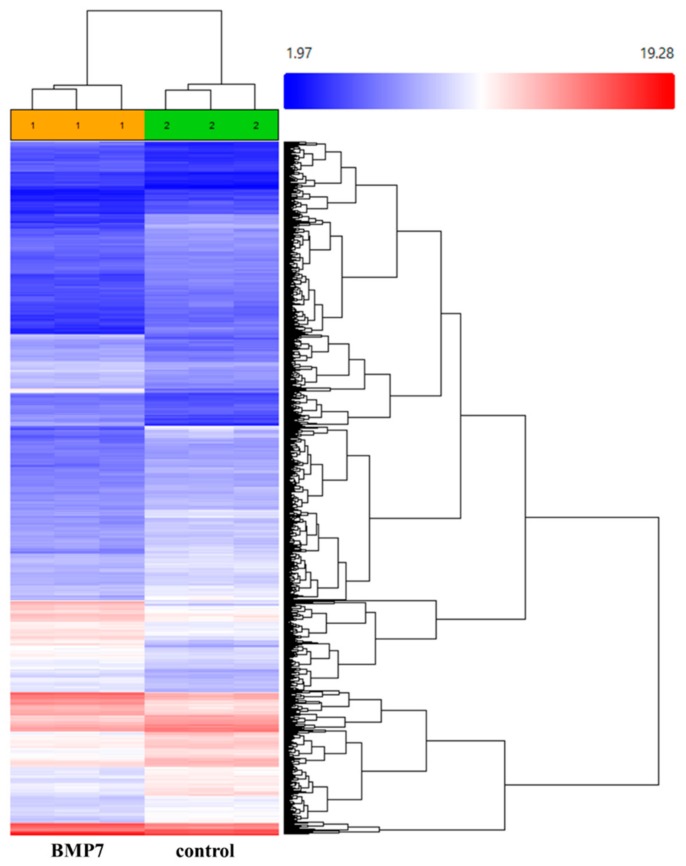
Hierarchal illustration of the differentially expressed genes in hTCEpi cells after treatment with BMP7 (100 ng/mL). Both twofold upregulated and downregulated genes were clustered. Untreated hTCEpi cells were served as control. After 24 h of treatment with BMP7, RNA was isolated and transcriptome-wide gene level expression profiling was performed using Clariom^™^ S arrays. Array scanning and data analysis were performed using Affymetrix^®^ Expression Console^™^ software (v.1.4.1) that provides signal estimation and quality control functionality for the expression arrays, and the Affymetrix^®^ Transcription Analysis Console (TAC) software (v.3.1), which performs statistical analysis and provides a list of differentially expressed genes. Expression level analysis was performed using the normalization method based on the processing algorithm called robust multi-array average (RMA) which was improved by Signal Space Transformation (SST-RMA, Affymetrix). Each array was performed in triplicates.

Since treatment of SP and NGF enhanced the expression levels of BMP7 mRNA, we also studied the consequences of BMP7 on epithelial cell function by performing GEP of BMP7 stimulated epithelial cells. After treatment of hTCEpi cells with BMP7 for 24 h, cells were collected to isolate RNA and gene level differential expression analysis was performed using Clariom™ S arrays. Of the 21,448 genes analyzed, 2026 genes were found to be differentially expressed (1257 genes were up-regulated and 769 genes were down-regulated) after treatment of hTCEpi cells in the presence of BMP7 comparing to the control untreated cells (Figure 2).

Graphical representation of the distribution of differentially expressed genes is shown in Figure S1. An up-regulation of SMAD family member 6 (SMAD6), a natural inhibitor of BMPs signaling [43], in BMP7 treated epithelial cells (56-fold) serves as a positive control in our present study. Gene ontology analysis confirms differential expression of 11 out of 18 genes that are responsive to BMP stimulus in this present study: up-regulation of desmoglein 4 (DSG4), SMAD6, SMAD7, SMAD9, inhibitor of DNA-binding 1 (ID1), BMP2, distal-less homeobox 5 (DLX5), proprotein convertase subtilisin/kexin Type 6 (PCSK6), ADAM metallopeptidase with thrombospondin type 1 motif 7 (ADAMTS7), SKI-like proto-oncogene (SKIL); and down-regulation of twisted gastrulation BMP signaling modulator 1 (TWSG1). The most significantly impacted pathways, after stimulation of corneal epithelial cells with BMP7, were TGF-β signaling pathway and cell cycle (with *p* < 0.05) (Table 1).

Biological processes related to cell cycle and its regulation, microtubule and intermediate filament cytoskeleton organization and tissue development are most significantly impacted along with other processes after BMP7 stimulation (Table 2) (analyzed using Advaita Bio’s iPathwayGuide; http://www.advaitabio.com/ipathwayguide). 

A complete list of genes with most profound differential expressions after BMP7 treatment compared to control in hTCEpi cells, with a cutoff of Anova *p*-value 0.001 and fold change 8 or −8, are given in Table 3.

### 2.3. qRT-PCR Validation of the Transcriptome Analysis Data

**Figure 3:**
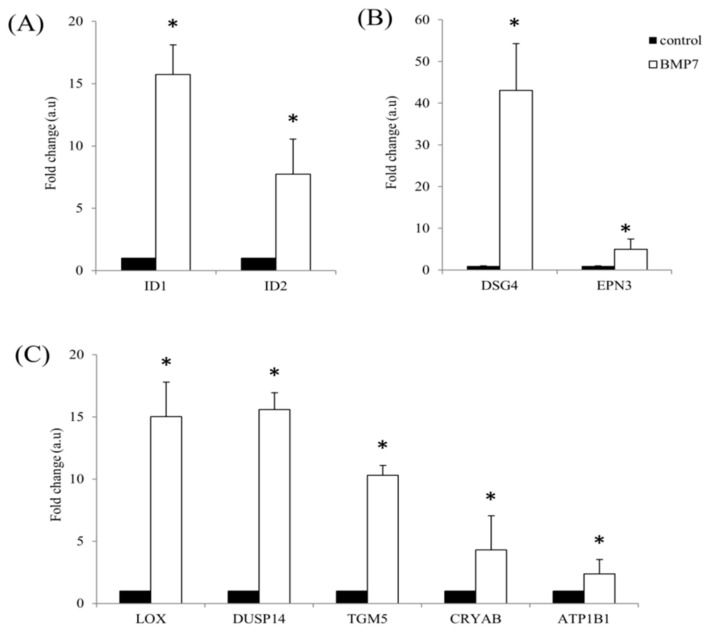
Validation of the Clariom^™^ S array identified differentially expressed transcripts. hTCEpi cells were cultured in the presence of 100 ng/mL BMP7 and RNA was isolated after 24 h. Reverse transcription was performed using 1 µg of total RNA to prepare cDNA and qRT-PCR was performed to find differentially expressed transcripts using respective primers. (**A**) ID proteins (ID1 and ID2); (**B**) cytoskeletal proteins (DSG4 and EPN3); and (**C**) metabolic enzymes (LOX, DUSP14, TGM5, CRYAB, and ATP1B1) differential expression was analyzed using respective primers. Relative expression pattern was analyzed by the comparative threshold cycle (2^−ΔΔ*C*t^) method. Expression values were represented as fold change over the control on an arbitrary scale after normalization with GAPDH. Data represent mean ± standard deviation of three independent experiments. *p*-values of <0.05 were considered statistically significant and are indicated by asterisks (*).

To further validate the transcriptome analysis data obtained after treatment of hTCEpi cells with BMP7, we performed qRT-PCR of some selected up-regulated transcripts using RNA isolated from BMP7 treated hTCEpi cells. Differential expression of various cytoskeletal proteins, ID proteins and metabolic enzymes were analyzed and confirmed. Microarray data of ID1 and ID2 shows an up-regulation of 37.6-fold and 8.3-fold, whereas qRT-PCR shows 15.7-fold and 7.7-fold. (Figure 3A). Of the analyzed cytoskeletal proteins DSG4 and epsin 3 (EPN3), microarray data show an up-regulation of 43.5-fold, 13.6-fold, whereas qRT-PCR data show an up-regulation of 43-fold and 5-fold (Figure 3B). Similarly, of the analyzed metabolic enzymes lysyl oxidase (LOX), dual specificity phosphatase 14 (DUSP14), transglutaminase 5 (TGM5), crystallin alpha B (CRYAB) and ATPase, Na^+^/K^+^ transporting, beta 1 (ATP1B1), microarray data show an up-regulation of 20-fold, 10-fold, 96-fold, 28-fold and 12.8-fold, whereas qRT-PCR data show 15-fold, 15.6-fold, 10-fold, 4.3-fold and 2.3-fold (Figure 3C). 

### 2.4. Influence of rhBMP7 on the Migration of hTCEpi Cells

**Figure 4:**
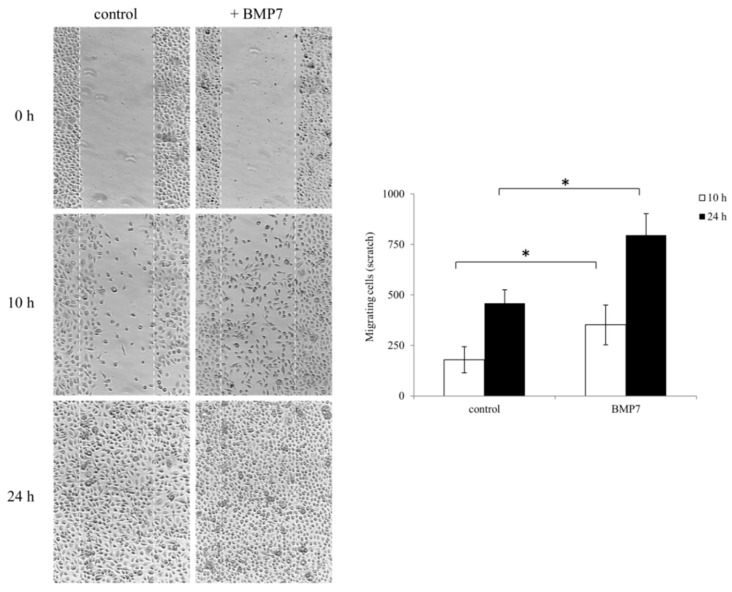
Wound healing scratch assay was made on 24 h growth factor-starved, confluent hTCEpi cells by scratching a line across the bottom of the culture dish. BMP7 was added (100 ng/mL) to the culture media and the cell motility and migration was observed at time points 10 and 24 h. The micrographs show the extent of scratch closure obtained under control conditions compared to those with the addition of BMP7. Cell migration quantification was evaluated by counting the number of cells in the central gap. Three independent experiments were performed and a representative result is shown. *p*-values of <0.05 were considered statistically significant and are indicated by asterisks (*).

Based on our GEP data, we further studied the effect of BMP7 on epithelial cell motility and migration by performing a scratch wound healing assay. We observed an enhanced motility and migration of epithelial cells in the presence of BMP7. During scratch assay, BMP7 treated corneal epithelial cells filled the scratch area much faster than untreated control cells. Cell migration was represented as number of cells filling the central gap area after making the scratch. We observed a significant increase in the number of migrating cells after BMP7 treatment in conditions, 10 and 24 h, after making the scratch compared to the control cells (Figure 4).

### 2.5. Stimulation of hTCEpi cells and Stromal Fibroblasts (SFs) by rhBMP7 Activates Vital Signaling Molecules

**Figure 5:**
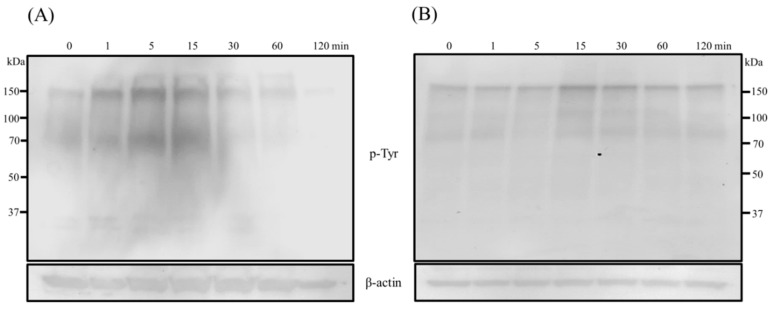
Protein tyrosine phosphorylation levels of (**A**) hTCEpi cells and (**B**) SFs after treatment with BMP7 (100 ng/mL) at different time points. Cells were treated with BMP7 and the total cell lysates were collected at time points 1, 5, 15, 30, 60 and 120 min, and subjected to immunoblot analysis. An increased total protein tyrosine phosphorylation (lasted until 30–60 min approximately) following BMP7 stimulation was evident. Amounts of the β-actin protein levels which were detected with an anti-β-actin antibody served as a loading control. One representative immunoblot of at least three independent experiments is shown.

Since the corneal stromal cells also plays an equivalent role along with epithelial cells during wound healing response, we also included SFs along with epithelial cells in the present study to investigate the influence of BMP7-mediated signaling events. Stimulation of hTCEpi cells and SFs with BMP7 resulted to the activation of cellular signal transduction cascades after the addition of BMP7. Increased protein tyrosine phosphorylation, an indispensable mechanism of signal transduction, was observed after the addition of BMP7 to both hTCEpi cells and SFs (Figure 5).

**Figure 6:**
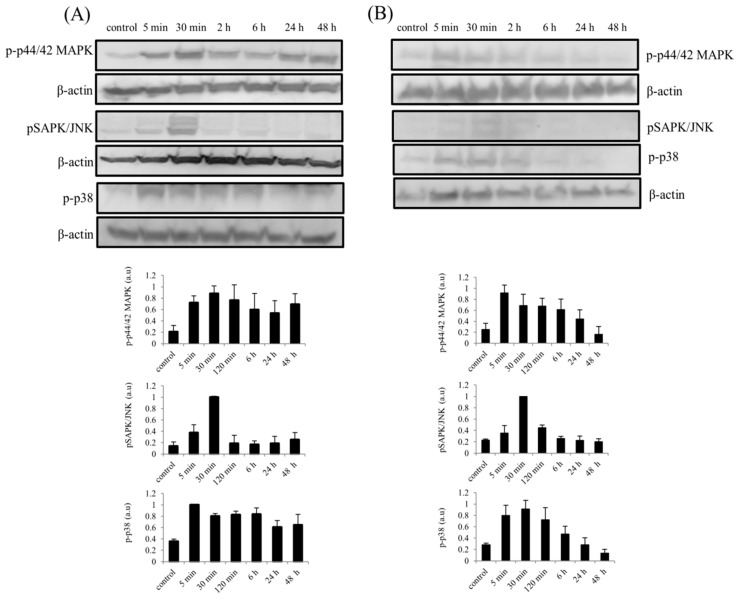
Activation of MAPK cascade proteins in (**A**) hTCEpi cells and (**B**) SFs after treatment with BMP7 (100 ng/mL) at different time points. Cells were treated with BMP7 and the total cell lysates were collected at time points 5 min, 30 min, 2, 6, 24 and 48 h after stimulation, and subjected to immunoblot analysis. Sustained activation of p44/42 MAPK and p38 until 48 h in hTCEpi cells and until 6 h in SFs was observed, whereas, in both cell types, only transient activation of pSAPK/JNK was observed. Amounts of the β-actin protein levels which were detected with an anti-β-actin antibody served as a loading control. Differences in the phosphorylation levels (p44/42 MAPK, SAPK/JNK and p38) were quantified using ImageJ software and the values were normalized to the corresponding β-actin signal. Data represent mean ± standard deviation of the phosphorylation levels shown as arbitrary units from more than three independent experiments. One representative immunoblot of at least three independent experiments is shown.

Similarly, an increase in the phosphorylation of mitogen-activated protein kinase (MAPK) cascade proteins (p44/42 MAPK, pSAPK/JNK and p38) was also observed in both hTCEpi cells and SFs. Phosphorylation of p44/42 MAPK (ERK1/2) and p38 was increased immediately, within 5 min, after stimulation of hTCEpi cells with BMP7, whereas phosphorylation of SAPK/JNK shows a gradual increase after 5–15 min of BMP7 stimulations and reaches its peak activation at 30 min (Figure 6A). Furthermore, sustained phosphorylation of p44/42 MAPK and p38 was observed until 48 h in BMP7 stimulated epithelial cells. Similar results were obtained after stimulation of hTCEpi cells with TGF-β1 (Figure S2). Treatment of epithelial cells in the presence of MEK1/MEK2 inhibitor, U0126 (10 µM) completely abolished phosphorylation of p44/42 MAPK (Figure S3A) and further confirms the activation of Raf/MEK/ERK pathway during BMP7 stimulation. Additionally, stimulation of corneal epithelial cells in the presence of BMP7 antagonist noggin increases phosphorylation of p44/42 MAPK (Figure S3B).

Studies corresponding to the effect of BMP7 on primary SFs also showed an increased activation of p44/42 MAPK after 5 min along with p38. Activation of p44/42 MAPK and p38 was observed to sustain until 6 h. An increased phosphorylation of SAPK/JNK was observed at 30 min after BMP stimulation which was transient until 2 h after stimulation (Figure 6B). 

**Figure 7:**
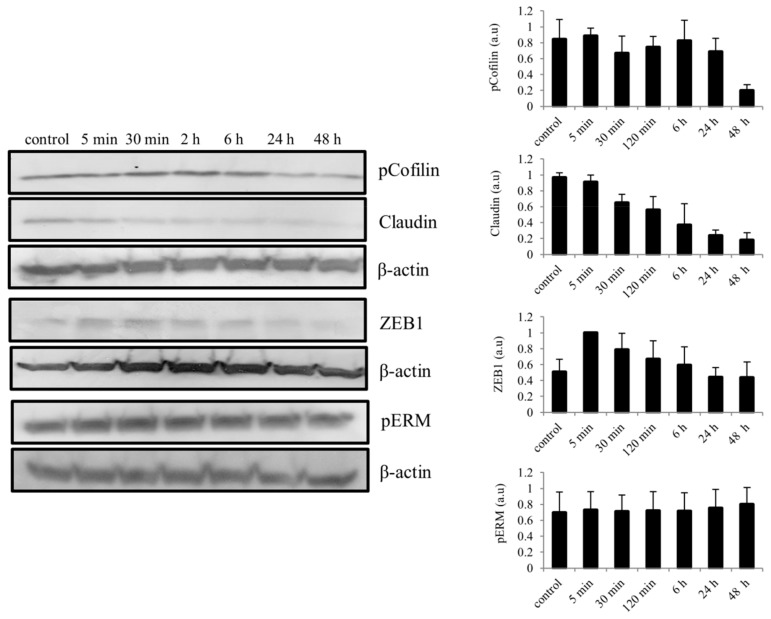
Activation of EMT-related signaling molecules in epithelial cells following the stimulation of BMP7 (100 ng/mL). Total protein lysates were collected at time points 5 min, 30 min, 2, 6, 24 and 48 h and subjected to immunoblot analysis. Differences in the phosphorylation levels (ERM and cofilin) and total protein levels (Zeb1 and claudin) was observed and quantified using ImageJ software and the values were normalized to the corresponding β-actin signal. Data represent mean ± standard deviation of the phosphorylation levels or total protein levels shown as arbitrary units from more than three independent experiments. One representative immunoblot of at least three independent experiments is shown.

As EMT-like phenomenon is also an important process during epithelial wound healing, we also considered to evaluate important activated EMT-related signaling molecules following BMP7 stimulation in hTCEpi cells. After BMP7 stimulation, differences in the phosphorylation levels of cofilin, a member of actin binding family, was observed which was decreased gradually by 48 h. Likewise, a decrease in the expression of claudin was also observed. Furthermore, we also observed an increase in the expression of transcription factor Zink finger E-box-binding homeobox 1 (ZEB1) immediately after stimulation with BMP7, which reaches its endogenous levels after 48 h of stimulation. A slight increase in the phosphorylation of ezrin/radixin/moesin (ERM) was also detected in epithelial cells upon stimulation with BMP7 (Figure 7).

**Figure 8:**
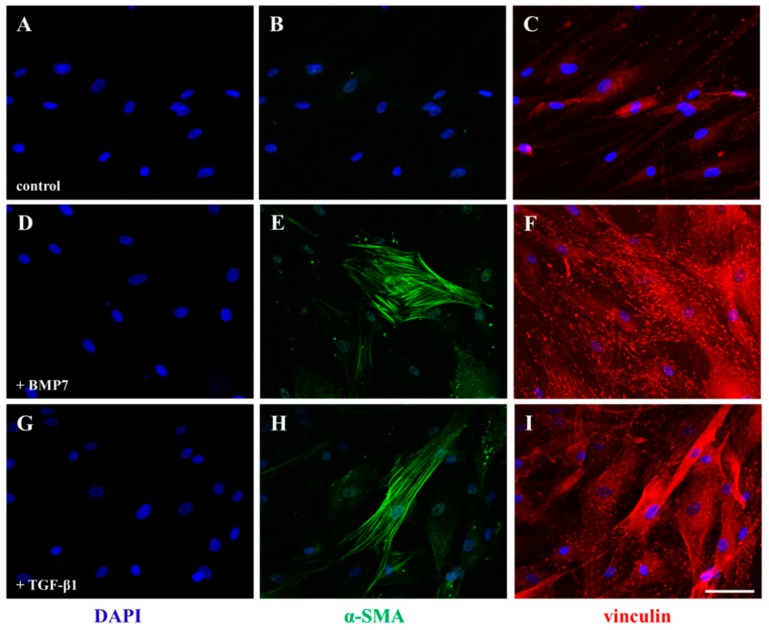
Immunofluorescence detection of the expression of α-SMA in primary human SFs after treatment with BMP7. Serum-starved SFs were cultured in the presence of BMP7 (100 ng/mL) and TGF-β1 (10 ng/mL) for 72 h and their activation was analyzed by staining with an anti-α-SMA (green) and vinculin (red) antibodies. An increase in the expression of focal adhesions was observed with vinculin antibody staining and the transformation of fibroblasts into activated myofibroblats was observed with α-SMA antibody staining. (**B**) (control), (**E**) (+BMP7) and (**H**) (+TGF-β1) represent α-SMA staining (green) and (**C**) (control), (**F**) (+BMP7) and (**I**) (+TGF-β1) represent vinculin (red) immune staining. (**A**,**D**,**G**) represent nuclei counter stained with 4,6-diamidino-2-phenylindole (DAPI) (blue). Scale bar: 50 μm.

Expression of α-smooth muscle actin (SMA) in stromal cells is an important phenomenon of transforming into repair phenotype during wound healing response. To study the role of BMP7 in transforming SFs into myofibroblasts, SFs were cultured in the presence of BMP7 and α-SMA expression was analyzed by immunocytochemistry. Figure 8A–C represent control SFs. We observed expression of α-SMA in SFs after 72 h treatment with BMP7 (Figure 8D,E) which indicates transformation of SFs into active myofibroblasts. Additionally, we also observed an increase in the vinculin stained focal adhesions, which regulate cell migration, upon stimulation with BMP7 (Figure 8F). TGF-β1 stimulated SFs served as a positive control (Figure 8G–I) in our experiments. Furthermore, in contrast to our observations in corneal epithelial cells, we observed decreased expression of phosphorylated ERM, Zeb1 expression and increased phosphorylation of cofilin in SFs following BMP7 stimulation after 24 h (Figure S4). 

### 2.6. Elevated Phosphorylation of Epidermal Growth Factor Receptor (EGFR) Following rhBMP7 Stimulation

**Figure 9:**
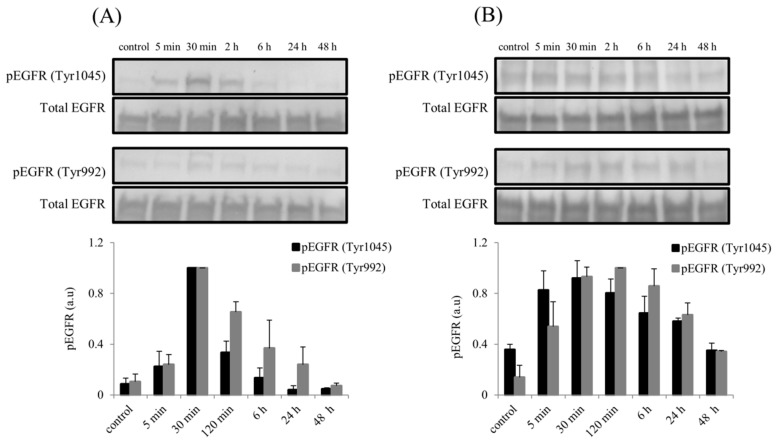
Activation of EGFR following BMP7 stimulation in (**A**) hTCEpi cells and (**B**) SFs. After BMP7 stimulation (100 ng/mL), total cell protein lysates were collected at time points 5 min, 30 min, 2, 6, 24, and 48 h and subjected to immunoblot analysis. Phosphorylation of EGFR at Tyr1045 and Tyr992 were analyzed using respective antibodies. Total EGFR protein levels were used to compare and calculate the differences in the phosphorylation levels. Differences in the phosphorylation levels (Tyr1045 and, Tyr992) were quantified using ImageJ software and the values were normalized to the corresponding total EGFR signal. Data represent mean ± standard deviation of the phosphorylation levels shown as arbitrary units from three independent experiments. One representative immunoblot of at least 3 independent experiments is shown.

Stimulation of hTCEpi cells and SFs with BMP7 also resulted to the activation of EGFR along with MAPK signaling pathway. Elevated phosphorylation of EGFR at Tyr1045 and at Tyr992 was observed immediately after BMP7 stimulation (Figure 9). In epithelial cells, phosphorylation of EGFR at Tyr1045 and Tyr992 reached its maximum by 30 min and decreased gradually to its normal endogenous levels after 24 h of stimulation (Figure 9A). In SFs, we observed sustained activation of EGFR at Tyr992 until 48 h of stimulation whereas EGFR at Tyr1045 reached its endogenous levels by 48 h of stimulation with BMP7 (Figure 9B). No differences were observed in the total EGFR protein levels during the course of BMP7 stimulation. Similarly, compared to the BMP7 stimulation alone, no difference in the phosphorylation of EGFR observed when the epithelial cells were stimulated with BMP7 in the presence of its antagonist noggin (Figure S3B). 

## 3. Discussion

In the human cornea, interactions between stromal and epithelial cells as well as epithelial and neuronal cells [44,45,46] play an important role in modulating the corneal function. In our previous study, we showed an increase in the expression of neuropeptide SP during corneal epithelial-trigeminal neuron interactions [40]. Similarly, NGF also produced constitutively in the human cornea by epithelial cells, SFs and modifies their functional activities [8,47]. Both SP and NGF are known to upregulate TGF-β1 mRNA and potentiate its production [6,7,48]. Since neuropeptides also regulate the corneal epithelial and stromal cell function [8,49,50,51], we also studied the effect of SP and NGF on the expression of BMP7 mRNA. Similar to the brain-derived neurotrophic factor induced expression of BMP7 [52], we also observed an enhanced BMP7 mRNA expression in corneal epithelial cells upon stimulation with SP and NGF whereas no such difference was observed in SFs. Thus, based on this observation along with the prominent role of BMP7 signaling in the modulation of cellular interactions [33], this study was designed to evaluate the effect of exogenous rhBMP7 on corneal epithelial and stromal cell function.

Transcriptome-wide gene-level expression profiling demonstrates relatively high number of differentially expressed genes as a result of rhBMP7 treatment on corneal epithelial cells. BMP7 treated corneal epithelial cells shows upregulation of TGF-β, cell cycle, JAK-STAT and MAPK signaling pathways. Further, gene ontology analysis predicts BMP7 involvement in the regulation of various vital biological processes such as regulation of cell cycle, cytoskeleton, intermediate filament and microtubule organization. BMP7 modulation of all these vital processes in corneal epithelial cells highlights its strong impact on epithelial cell function. In our study, of all differentially expressed BMP7 responsive genes, only TWSG1 was found to be downregulated. TWSG1 functions as an antagonist to BMPs [53] and its downregulation suggests a stimulatory function for BMP signaling [54]. In this context, its downregulation in corneal epithelial cells further confirms the activation of BMP signaling pathway. Among the various BMP target genes, we observed activation of ID1, ID2, ID3, FOXN1, SMAD6, SMAD7, DUSP1, BAMBI, BMP2, and MSX2 genes [55,56,57] after stimulation of corneal epithelial cells with BMP7. Subsequently, different DUSPs are also observed to be upregulated in our study similar to the treatment of BMP6 [55]. Epsin 3 is known to serve an important function in activated epithelial cells during tissue morphogenesis [58] and BMP7 stimulated expression of Epsin 3 in corneal epithelial cells may also help in modulating the activation of epithelial cells. Furthermore, LOX, a copper-containing amine oxidase which is known to be upregulated by BMP2/4 [59], is also upregulated upon BMP7 stimulation of corneal epithelial cells. Since LOX is also an inducer of EMT-like response [60,61], its upregulation denotes possible involvement of BMP7 in EMT-like phenomenon of corneal epithelial cells.

During BMP7 stimulation of corneal epithelial cells, we observed changes in some of the actin cytoskeleton regulating molecules and EMT-related proteins. Cofilin is an actin binding phosphoprotein and different cellular responses alter its phosphorylation levels [62,63]. Phosphorylation inactivates cofilin and cells require dephosphorylated cofilin to induce actin assembly and lamellipodium extension to ensue cell motility [64,65]. The observed gradual dephosphorylation of cofilin after BMP7 stimulation may activates cofilin and plays crucial role in the regulation of actin cytoskeleton remodeling, an important determinant of cell motility. By linking actin filaments to plasma membrane, ERM proteins involved in cell–cell adhesion, cytoskeleton organization as well as in signaling [66]. Furthermore, ERM protein phosphorylation is shown to be important for cell movement [67,68] and the observed small increase in the phosphorylation levels following BMP7 stimulation may contribute to the motility of cells. Additionally, tight junction (TJ) protein claudin also functions as a motility molecule [69] and its decreased expression enhances cell migration [70] in certain cell-types. Similarly, during the initiation of EMT, disrupted TJs lead to the reduced expression of claudin and other TJ proteins [71,72]. The observed decline in the claudin expression levels following BMP7 stimulation, along with decreased phosphorylation of cofilin, may also contribute to enhance cell migration and motility in corneal epithelial cells. Apart from the progression of EMT [72], Zeb1 is also participates in regulation of cell proliferation [73,74] and its expression is known to be induced by TGF-β and growth factor activated MAPK signaling pathways [75,76,77]. Similar to these reports, the observed increase in Zeb1 expression after BMP7 stimulation accompanied by increased MAPK pathway may correlate cell proliferation and EMT-like phenomenon in our study. 

Adaptation of cells from a sedentary to the migratory phenotype is an important process of EMT [78]. Activation of MAPK cascade components (ERK1/2, JNK and p38) is essential for the movement of corneal epithelial cells [79,80,81] as well as EMT [82]. Additionally, BMP7 stimulation also leads to the activation of MAPK pathway [38,83,84] and is involved in both progression [38] as well as reversal of epithelial-to-mesenchymal transition (EMT)-like phenomenon [85]. Upon stimulation with BMP7, corneal epithelial cells show persistent activation of MAPK cascade proteins p44/42 MAPK and p38 up to 48 h, whereas JNK was activated only transiently. Since it is known that activated MAPK cascades facilitate expression of EMT-associated transcription factors [72], BMP7 induced MAPK proteins also may participate in the initiation of EMT-like responses. Taking together, our results along with literature reports support the notion that BMP7 may play an important role in the EMT-like phenomenon during corneal epithelial cell migration. 

During corneal tissue repair, stimulation by growth factors activates p44/42 MAPK and p38 proteins, but not of JNK, and their cross talk is essential to synchronize the dynamics of wound healing process [86]. Accordingly, we also found activated p44/42 MAPK and p38 pathways following BMP7 stimulation in corneal epithelial cells and SFs. Activation of JNK was only transient following BMP7 stimulation and reaches its endogenous levels. Generally, in the corneal cells JNK is activated under stress-related circumstances [87,88] and inhibition of its activation potentially increases the damage repair response [88]. Furthermore, since JNK is also known as stress-activated protein kinase and its activation is dependent mainly on the type of stimulation [89], in our study, signaling events initiated by BMP7 stimulation may drive cell survival mechanisms by suppressing the activation of JNK and thereby enhance repair process. Similarly, stimulation of p44/42 MAPK and p38 is necessary for the α-SMA expression and transformation of fibroblasts into myofibroblasts [90,91,92,93,94]. In the present study, the observed activation of p44/42 MAPK and p38 following stimulation of SFs with BMP7 along with the expression of α-SMA may also suggest the involvement of activated MAPK cascade proteins in the differentiation of SFs into myofibroblasts. 

Another important aspect of the present study is documentation of the cross talk between BMP7 and EGFR signaling. EGFR, with its intrinsic tyrosine kinase activity, triggers signaling pathways related to migration, proliferation and EMT [95]. EGFR serves as a predominant receptor for multiple ligands and is activated by endogenous growth factors such as EGF and TGF-α [96]. Activated EGFR plays an important role in the corneal epithelial cell and SFs proliferation and migration [97,98]. Correspondingly, in the present study, during stimulation with BMP7, EGFR was activated, phosphorylated at Tyr1045 and Tyr992 without any change in the total EGFR protein levels and its phosphorylation levels remain elevated for as long as 24 h. BMP7 regulation of EGFR was also reported in hepatic stellate cells [29] and astrocytes [99] which can modulate different cellular functions. Since EGFR-mediated signals reported to also activate MAPK cascades and thereby regulate diverse cellular functions [96,100], the ongoing cross talk between BMP7 and EGFR may have a potential role in corneal epithelial cell function and may enhance their migration. The documented augmentation of epithelial proliferation and migration after treatment with BMP7 could also reflect the synergistic consequence of activated EGFR. EGFR phosphorylation at Tyr992 acts as a high-affinity binding site for phospholipase Cγ and also activates MAPK cascade [101], whereas phosphorylation of EGFR at Tyr1045 serves as a binding site for c-Cbl which inhibits EGFR signaling by inducing receptor degradation [102]. Moreover, activation of EGFR by TGF family members results in receptor recycling [96] as well as MAPK activation [103]. Nevertheless, because of the contrasting implications of EGFR phosphorylation at Tyr1045 as well as Tyr992, and since BMP7 phosphorylates EGFR at both tyrosine residues, it is challenging to conclude the outcome of BMP7 stimulated EGFR activation in corneal epithelial cells and SFs. Furthermore, as the activated EGFR stimulates signaling events necessary for the generation of cell contractile force [104,105] through MAPK pathway [106,107] the observed activation of EGFR and MAPK signaling by BMP7 may enhance cell motility and migration during wound healing in both corneal epithelial cells and SFs. 

Even though we did not observe any difference in the BMP7 expression by SP and NGF in SFs, they still respond to BMP7 stimulation in a manner similar to epithelial cells indicating a role for BMP7-mediated pathways in wound healing. Similar to the corneal epithelial cells, primary SFs also show an increase in the tyrosine phosphorylation following BMP7 stimulation. SFs display characteristics similar to smooth muscle cells and transform into myofibroblasts by expressing focal adhesions in the presence of TGF-β [108]. Similarly, the observed increase in the expression of α-SMA and focal adhesions upon BMP7 stimulation shows that BMP7 mimics the role of TGF-β in SFs which may also assist in increasing their contractile activity [109]. Furthermore, BMP7 induced expression of α-SMA is also reported in hepatic stellate cells [110]. This is in contrast to the in vivo observations where the presence of BMP7 suppressed expression levels of α-SMA [35,36]. In general, cellular response to growth regulating cytokines is influenced by the density of cells [111], which further affect the number of receptors present on their surface. A decrease in receptor number with an increase in cell density was observed for EGF [112], TNF [111] and TGF-β [113] receptors. Since BMP7 gene expression also resulted to the increased cell proliferation in vivo, occurrence of density-dependent reduction in BMP7 receptors may also happen due to the increased cell density, which may lead to the reduced BMP7 signaling and decreased expression of α-SMA in contrast to our observation of increased α-SMA expression in vitro. An increase in the levels of vinculin [108], a major component of the focal adhesions, following BMP7 treatment further shows the activated state of SFs and supports our assumption. Furthermore, since epithelial cell injury augments SFs myodifferentiation by increasing the secretion of TGF-β [114], the observed increase of BMP7 mRNA in corneal epithelial cells and BMP7 stimulated α-SMA expression in SFs may also play a vital role during corneal wound healing response. 

Taken together, our results indicate a close correlation between the actions of TGF-β and BMP7 in corneal epithelial and stromal cell function. Both cytokines belongs to the same TGF-β superfamily and share similar downstream canonical Smad-dependent signaling pathways [115], display similar gene expression profiles [116], and are known to induce EMT. Our transcriptome analysis data also revealed the activation of TGF-β signaling pathway in corneal epithelial cells following the addition of BMP7. TGF-β mediated increased expression of α-SMA transform fibroblasts into activated myofibroblasts. Similarly, in this study, we also showed BMP7 mediated expression of α-SMA in SFs which may activate and transform them into myofibroblasts. TGF-β can induce activation of MAPK cascades, transactivation of EGFR [27,72], and we also demonstrated the activation of MAPK cascade upon BMP7 stimulation in corneal epithelial cells and SFs along with transactivation of EGFR. TGF-β1-mediated transactivation of EGFR occurs during corneal stromal cell differentiation [30] and we also showed the EGFR transactivation by BMP7 in SFs. Similar to the regulation of TGF-β, BMP7 expression was also regulated by SP and NGF. Furthermore, BMP7- mediated cellular responses that were observed in the present study are shown in Figure S5. In the corneal epithelial cells, BMP7 stimulated consequences including increased cell migration, increased Zeb1 levels, decreased claudin expression, activation of MAPK cascades and increased LOX expression were also reported to be EMT-like responses. Based on the results presented in this study, it is likely that BMP7 may also contribute to EMT-like responses and play a role equivalent to TGF-β during the course of corneal tissue repair. Furthermore, our transcriptome analysis data on corneal epithelial cells can be helpful to comprehend differentially expressed genes and activated pathways and may contribute to the development of novel therapeutic targets. 

## 4. Materials and Methods

### 4.1. Materials

The following antibodies used in the present study were purchased from Cell Signaling Technology (Frankfurt, Germany): EGF receptor (Cat No. 4267), phospho-EGF receptor (Tyr1068) (Cat No. 3777), phospho-EGF receptor (Tyr992) (Cat No. 2235), phospho-tyrosine (Cat No. 9411), β-Actin (Cat No. 4970), phospho-SAPK/JNK (Cat No. 4668), phospho-p38 MAPK (Cat No. 4511), phospho-p44/42 MAPK (Cat No. 4370), claudin-1 (Cat No. 13255), phospho-cofilin (Cat No. 3313), TCF8/ZEB1 (Cat No. 3396) and phospho-ERM (Cat No. 3726). Alpha-SMA (Cat No. ab5694) was from Abcam, Cambridge, UK. Vinculin (Cat No. V9131) antibody was from Sigma-Aldrich (Munich, Germany). Anti-rabbit IgG- HRP antibody (Cat No. 7074) and anti-mouse IgG-HRP antibody (Cat No. 7076) were purchased from Cell Signaling Technology (Frankfurt, Germany). TPP tissue culture flasks, dishes and 6- and 12-well plates were obtained from Sigma-Aldrich (Munich, Germany). Coverslips and glass slides were from Marienfeld (Bonn, Germany). ECL prime detection western blotting reagent was purchased from Amersham and other western blotting reagents were from Bio-Rad (Munich, Germany). SP was purchased from Tocris (Wiesbaden-Nordenstadt, Germany). rh BMP7 (Cat No. 11343293) and rh TGF-β1 (Cat No. 11343161), rh NGF (Cat No. 11343354) were purchased from ImmunoTools (Friesoythe, Germany). rh noggin (Cat No. SRP4675) was purchased from Sigma-Aldrich (Munich, Germany). QuantiTect Reverse Transcription kit was purchased from Qiagen (Hilden, Germany), and the innuMIX qPCR MasterMix SyGreen was from Analytik-Jena (Jena, Germany). Primers were synthesized from Metabion GmbH (Martinsried, Germany). U0126 (Cat No. 9903) was purchased from Cell Signaling Technology (Frankfurt, Germany). 

### 4.2. Cell Culture

This study was approved by the ethics committee of the University of Rostock and followed the guidelines of the Declaration of Helsinki. 

Telomerase-immortalized human corneal epithelial cell line [117] (a kind gift of Prof. J. V. Jester, University of California, Irvine, CA, USA; authenticated and characterized according to ATCC standard protocols), between passages 22 and 30, was used in this present study. The hTCEpi cells were cultured in KGM-Gold™ growth medium (Lonza, Köln, Germany). Cells were sub-cultured on T75 tissue culture flasks (Sigma-Aldrich, Munich, Germany), incubated at 37 °C in 5% CO_2_ and passaged every 5–7 days.

Primary human SFs were cultured using an explant culture method, in DMEM with low glucose (Sigma-Aldrich, Munich, Germany) supplemented with 10% FCS, after collecting corneas from donor cadavers. SFs were seen growing out of the corneal explants after 3–4 days. When outgrowing primary SFs reached a confluent monolayer, cells were trypsinized and sub-cultured. For experimental analysis, SFs of third passage was used. The mesenchymal origin of the cells was confirmed by vimentin antibody immune staining.

In the present study, cells were treated with 100 ng/mL of rh BMP7 [118,119,120], which is less than the concentration of BMP7 used in other studies on corneal keratocytes and epithelial cells [34,121]. No toxic effects on epithelial cells and SFs were observed while using rhBMP7. TGF-β1 expression is known to be increased during injury in cultured SFs [122], which further helps SFs to differentiate into myofibroblasts [123]. Due to this reason, we also used TGF-β1 isoform at the concentration of 10 ng/mL in this present study. During stimulation of cells with BMP7 or TGF-β1, cells were growth factor- or serum-starved for 24 h before stimulation and cell culture media deprived of growth factors or serum was used. SP and NGF were used at the concentrations of 10^−5^ M and 100 ng/mL.

### 4.3. Microarray Hybridization

The cells were lysed in RTL Plus buffer, part of the RNeasy Plus Kit (Qiagen, Hilden, Germany) and extracted according to the manufacturers protocol. The whole RNA samples were quantified spectrophotometrically (NanoDrop, Thermo Fisher, Darmstadt, Germany) and the integrity was controlled using the Agilent Bioanalyzer 2100 with the RNA Nano chip kit (Agilent Genomics, Waldbronn, Germany). RNA integrity number values between 9.0 and 9.8 were achieved and 200 ng were used as starting material. To perform the Expression Profiling, Affymetrix Clariom^TM^ S Arrays were used according to the manufacturer’s instructions (Affymetrix, St. Clara, CA, USA). The hybridization was carried out overnight at 45 °C in the GeneChip^®^ Hybridisation Oven 645 (Affymetrix). The microarray was scanned using the GeneChip Scanner 3000 (Affymetrix) at 0.7 micron resolution. 

### 4.4. Quantitative Real-Time Polymerase Chain Reaction (qRT-PCR)

Total RNA was isolated from cultured corneal epithelial cells or SFs using the NucleoSpin^®^ RNA kit (Macherey-Nagel, Berlin, Germany) according to the manufacturer’s instructions. Single-stranded cDNA was prepared with 1 µg of total RNA using QuantiTect Reverse Transcription kit (Qiagen, Hilden, Germany). Intron/exon spanning primers were designed from the respective GenBank sequences using VectorNTI software (Invitrogen) based on minimal hairpin, duplex formation and guanine cytosine composition. List of primers used in this study is given in Table 4.

The synthesized cDNA then was used as a template for qRT-PCR using the innuMIX qPCR MasterMix SyGreen and qTower 2.0 (Analytik Jena, Jena, Germany). The cycling conditions used for amplification were 95 °C for 2 min, 40 cycles of 95 °C for 5 s and 65 °C for 25 s. Later, the expression of all genes was normalized to the expression of the corresponding housekeeping gene, GAPDH. The relative amount of target mRNA in the unstimulated control cells and BMP7 treated cells was analyzed using the ΔΔ*C*t method, as described previously [124].

### 4.5. Immunofluorescence

Human primary SFs grown on glass coverslips were fixed with 4% paraformaldehyde for 10 min after washing with PBS and then permeabilized with PBS containing 0.1% Triton X-100 (Sigma-Aldrich, Munich, Germany) for 30 min. Fixed and permeabilized cells were then incubated with primary antibodies for intracellular staining. All primary antibodies in this study were used at 1:100 dilutions in PBS containing 0.1% Triton X-100 with 2% FCS and incubated for 60 min at room temperature. The cells were then washed with PBS before adding secondary donkey anti-mouse IgG (H+L)-Alexa Fluor 488 (diluted 1:50) or donkey anti-rabbit IgG (H+L)-Cy3 (diluted 1:100) and incubated at room temperature for another 60 min. Later, cells were washed 3 times with PBS and mounted in mounting medium (Vector Labs, Eching, Germany) containing 4,6-diamidino-2-phenylindole (DAPI). During immunostaining, cells stained with only secondary antibodies and did not show any specific staining served as a control. Cells were observed under a Nikon confocal fluorescence microscope equipped with digital camera (Nikon Eclipse E400 with D-Eclipse C1) and all images were taken from a single plane through the cell monolayers with 40× objective using the same settings.

### 4.6. Immunoblotting

Corneal epithelial or SFs monolayers were washed with PBS and lysed in RIPA buffer (Sigma-Aldrich, Munich, Germany) containing protease and phosphatase inhibitors (Roche, Mannheim, Germany). Equal amounts of total cell lysates (30 µg protein per lane) were loaded into the wells and separated by SDS-PAGE using 10% Mini-PROTEAN^®^ TGX™ precast gels (Bio-Rad, Munich, Germany) and transferred onto PVDF membranes (Bio-Rad, Munich, Germany). Later, membranes were blocked with 5% non-fat dry milk (Carl Roth, Karlsruhe, Germany) in Tris-buffered saline with Tween-20 (Sigma-Aldrich, Munich, Germany) (TBS-T) for 30 min and incubated with respective primary antibodies (diluted 1:1000) overnight at 4 °C. After washing 3 times with TBS-T, membranes were incubated with secondary HRP-conjugated anti-rabbit or anti-mouse IgG (diluted 1:2500) for an additional 1 h at room temperature and developed to visualize protein bands using the enhanced chemiluminescence detection system (Amersham). During quantification, optical density of each protein band was normalized to the corresponding β-actin or GAPDH band. Quantification of the blots was performed using ImageJ software [125].

### 4.7. Scratch Assay

Migration of corneal epithelial cells was assessed by performing an in vitro scratch assay in which a linear scratch midline was made across the bottom of the dish on a confluent monolayer of epithelial cells using a 200 µL sterile pipet tip. After that, cells were rinsed gently with PBS to remove any remaining cell debris. KGM-Gold™ medium deprived of growth factors was used during the scratch assay. BMP7 was used at a concentration as mentioned earlier with fresh medium. Later, pictures were taken at 10× magnifications using a microscope equipped with Moticam10 digital camera at time points 10 and 24 h. Epithelial cell migration across the scratch line was then quantified by counting the cells invading the central scratch area in either the control or the BMP7 treated culture dish. The cell number was later represented for both conditions in each sample. 

### 4.8. Statistical Analyses

Bar charts and line plots were generated using means and the standard deviation. Student’s t-test was used for the comparison between two groups and *p*-values of <0.05 were considered statistically significant and are indicated by asterisks. 

## Figures and Tables

**Table 1:** Pathways significantly impacted during BMP7 stimulation in hTCEpi cells.

Pathway Name	*p*-Value	KEGG
TGF-β signaling pathway	5.780 × 10^−5^	KEGG: 04350
Cell cycle	2.556 × 10^−4^	KEGG: 04110
Jak-STAT signaling pathway	0.018	KEGG: 04630
MAPK signaling pathway	0.040	KEGG: 04010
Osteoclast differentiation	0.042	KEGG: 04380

Significantly impacted pathways after BMP stimulation (*p* < 0.05) were arranged according to the *p*-value. KEGG = Kyoto Encyclopedia of Genes and Genomes. The GEP data were analyzed using Advaita Bio’s iPathwayGuide (http://www.advaitabio.com/ipathwayguide).

**Table 2:** Biological processes significantly impacted during BMP7 stimulation in hTCEpi cells.

Biological Processes	Differentially Expressed Genes/Total Genes	GO Accession	*p*-Value
Mitotic cell cycle	111/216	GO: 0000278	4.000 × 10^−9^
Cell cycle	149/321	GO: 0007049	5.300 × 10^−8^
Cytoskeleton organization	82/153	GO: 0007010	5.500 × 10^−8^
Intermediate filament cytoskeleton organization	10/10	GO: 0045104	1.600 × 10^−5^
Regulation of cell cycle	82/171	GO: 0051726	2.300 × 10^−5^
Microtubule cytoskeleton organization	42/73	GO: 0000226	1.300 × 10^−5^
Intermediate filament organization	8/8	GO: 0045109	1.500× 10^−4^
Developmental process	252/655	GO: 0032502	3.100× 10^−4^
Tissue development	100/234	GO: 0009888	8.300 × 10^−4^
Negative regulation of protein phosphorylation	26/46	GO: 0001933	9.000 × 10^−4^
Cell proliferation	111/272	GO: 0008283	0.003
Epithelium development	67/153	GO: 0060429	0.003
TGF-β receptor signaling pathway	11/16	GO: 0007179	0.004
Inactivation of MAPK activity	5/5	GO: 0000188	0.004
Regulation of microtubule cytoskeleton organization	13/21	GO: 0070507	0.007
Positive regulation of Wnt signaling pathway	12/19	GO: 0030177	0.007
Positive regulation of cytoskeleton organization	26/52	GO: 0051493	0.009
MAPK cascade	41/90	GO: 0000165	0.009
Signal transduction by protein phosphorylation	42/93	GO: 0023014	0.010
Endothelial cell development	4/4	GO: 0001885	0.012
Desmosome organization	4/4	GO: 0002934	0.012
SMAD protein complex assembly	4/4	GO: 0007183	0.012
Osteoblast proliferation	4/4	GO: 0033687	0.012
Regulation of protein serine/threonine kinase activity	31/66	GO: 0071900	0.013
Cell surface receptor signaling pathway	102/258	GO: 0007166	0.014
Fibroblast proliferation	10/16	GO: 0048144	0.016
SMAD protein signal transduction	5/6	GO: 0060395	0.018
Regulation of actin polymerization or depolymerization	8/12	GO: 0008064	0.018
Regulation of phosphorylation	68/166	GO: 0042325	0.019
VEGF production	7/10	GO: 0010573	0.019
Interleukin-2 production	7/10	GO: 0032623	0.019
Cell adhesion	79/197	GO: 0007155	0.021
Epithelial cell differentiation	41/94	GO: 0030855	0.021
Response to BMP	11/18	GO: 0071772	0.024
Regulation of ROS metabolic process	14/26	GO: 2000377	0.024
Vesicle mediated transport	51/122	GO: 0016192	0.027
Chemokine production	9/15	GO: 0032602	0.030
Cell migration	62/153	GO: 0016477	0.031
Cellular response to TGF-β stimulus	14/27	GO: 0071560	0.035
Lipoxygenase pathway	3/3	GO: 0019372	0.037
Intermediate filament bundle assembly	3/3	GO: 0045110	0.037
Regulation of TGF-β receptor signaling pathway	7/11	GO: 0017015	0.038
Cell differentiation	151/407	GO: 0030154	0.039
Regulation of Interleukin-2 production	6/9	GO: 0032663	0.042
Response to growth factor	34/79	GO: 0070848	0.042
BMP signaling pathway	10/18	GO: 0030509	0.043
ATP generation from ADP	5/7	GO: 0006757	0.045
SMAD protein import to nucleus	4/5	GO: 0007184	0.045
Regulation of cyclin dependent protein kinase activity	12/23	GO: 1904029	0.047
Regeneration	18/38	GO: 0031099	0.049

Significantly impacted biological processes after BMP stimulation were arranged according to the *p*-value (*p* < 0.05). Differentially expressed genes in relation to the total number of genes in that process were denoted along with the gene ontology (GO) identifier. The GEP data was analyzed using Advaita Bio’s iPathwayGuide (http://www.advaitabio.com/ipathwayguide).

**Table 3:** List of genes with most profound differential expression after BMP7 treatment.

Fold Change (Down Regulated)	*p*-Value	Gene Symbol	Fold Change (Up Regulated)	*p*-Value	Gene Symbol
−64.32	0.00018	*HIST1H3G*	8.05	0.000406	*ALOX12B*
−58.47	0.000071	*TOP2A*	8.36	0.000067	*BAMBI*
−46.84	0.000005	*ANLN*	8.37	0.000013	*ID2*
−42.33	0.000046	*TFPI2*	8.39	0.000018	*LCE3E*
−41.85	0.000309	*HIST2H3A*	8.46	0.000245	*BDNF*
−37.77	0.000009	*NCAPG*	8.49	0.000242	*DLX3*
−36.57	0.000029	*CCNA2*	8.51	0.000018	*FAM46B*
−36.48	0.000107	*RRM2*	8.54	0.00008	*CGB2*
−34.74	0.000308	*HIST2H3A; HIST2H3C*	8.78	0.000024	*HK2*
−29.49	0.00016	*TTK*	8.83	0.000403	*FPR3*
−27.57	0.000001	*FAM111B*	8.84	0.00021	*BMP2*
−25.32	7.63 × 10^−7^	*NUSAP1*	9	0.000011	*CHRNA3*
−24.3	0.000036	*PLK1*	9.3	0.000094	*ULK3*
−20.9	0.000371	*DLGAP5*	9.58	0.000057	*DUSP1*
−19.5	0.000003	*MKI67*	9.6	0.000018	*GCOM1*
−16.79	0.000208	*IL1B*	9.74	0.000781	*GABRA3*
−16.57	0.000092	*CCNB2*	9.74	0.000021	*SDR9C7*
−16.55	0.000032	*ASPM*	9.74	0.000281	*RNF39*
−16.27	0.000055	*KIF18A*	9.81	0.000849	*CYP4F22*
−16.25	0.000072	*DTL*	9.92	0.00001	*DUSP14*
−15.81	0.000586	*HIST1H1B*	10.18	0.000032	*MXD1*
−15.57	0.00003	*GPR50*	10.34	0.00026	*ST3GAL4*
−15.31	0.000043	*CENPF*	10.44	0.000002	*SPOPL*
−15.23	0.000017	*SHCBP1*	10.58	0.000063	*SLC7A8*
−14.83	0.000305	*PBK*	10.67	0.00079	*LCE3C*
−14.71	0.000777	*CKAP2L*	10.72	0.000034	*PSCA*
−14.61	0.00001	*KIF20A*	10.89	0.000199	*DLX2*
−14.2	0.000094	*LMNB1*	11.02	0.000084	*LCE3D*
−14.13	0.000024	*MELK*	11.17	0.000127	*PLBD1*
−13.16	0.000482	*PRC1*	12.56	0.000338	*KPRP*
−12.65	0.000038	*DEPDC1*	12.88	0.000074	*ATP1B1*
−12.56	0.000045	*TACC3*	13.24	0.000107	*CGB1*
−12.53	0.000212	*HELLS*	13.68	0.000006	*EPN3*
−12.45	0.000029	*NCAPG2*	14.25	0.000003	*C10orf99*
−12.25	0.000355	*BUB1*	14.57	0.000011	*C5orf46*
−12.06	0.000473	*CASC5*	14.65	0.000002	*BPGM*
−11.89	0.000104	*NDC1*	14.84	0.000014	*GNA14*
−11.81	0.000635	*FAM83D*	14.93	0.000294	*C1QTNF3-AMACR*
−11.8	0.000488	*CCNE2*	14.96	0.000013	*CGB5; CGB8*
−11.43	0.000527	*KIF23*	14.97	0.000096	*SERPINB12*
−11.17	0.000167	*CDC20*	15.66	0.000279	*MARCH3*
−11.1	0.000901	*H2AFX*	16.07	3.57 × 10^−7^	*VSIG8*
−10.97	0.000061	*KIF2C*	16.67	0.000159	*USP2*
−10.77	0.000126	*STIL*	17.7	0.000147	*SLAMF9*
−10.75	0.000582	*NCAPH*	18.03	0.000376	*SPAG17*
−10.57	0.000444	*FOXM1*	18.8	0.000024	*CGB8*
−10.23	0.00036	*CCNB1*	18.93	0.000319	*CLDN17*
−10.09	0.000184	*TPX2*	18.98	0.000083	*NPR3*
−10.06	0.000045	*MCM5*	19.8	0.000229	*CGB; CGB5*
−9.91	0.000196	*XRCC2*	19.94	0.000024	*LOX*
−9.78	0.000011	*KIF14*	20	0.000026	*HPGD*
−9.44	0.000053	*ARHGAP11A*	26.9	0.000814	*SPINK7*
−9.4	0.000046	*DIAPH3*	28.3	0.000024	*CRYAB*
−9.26	0.000008	*MCM3*	29.87	0.000029	*DIO2*
−8.97	0.000141	*CDC6*	37.64	0.000037	*ID1*
−8.93	0.000088	*ARHGAP11B*	43.55	3.21 × 10^−7^	*DSG4*
−8.73	0.000689	*SGOL1*	56.48	0.000029	*SMAD6*
−8.61	0.00009	*FANCA*	64.3	0.000012	*AMACR*
−8.5	0.000047	*HJURP*	96.26	0.000007	*TGM5*
−8.39	0.000032	*ADORA2B*	98.81	0.000061	*SPINK6*
−8.01	0.000659	*TMEM97*	135.1	9.72 × 10^−7^	*FABP4*

Genes with most profound differential expressions after BMP7 treatment compared to control in hTCEpi cells (Filters used: Anova *p*-value 0.001 and fold change 8 or −8, respectively).

**Table 4:** List of primers used in the present study.

Target	Primer	Primer Sequence (5’-3’)	GenBank Accession
ATP1B1	fwd	TGAATTTAAGCCCACATATCAGGACCG	NM_001677
	rev	CATGTCATCCCTCTGGGCTGAATCT	
CRYAB	fwd	GGGAGATGTGATTGAGGTGCATGG	NM_001289807
	rev	AGGACCCCATCAGATGACAGGGAT	
DSG4	fwd	GCAGCCTGTCGAGAAGGAGAGGACA	NM_001134453
	rev	GTGATGTTAATTTCCCCAGTGCGAGG	
EPN3	fwd	CTGCGCCTGAGCCGGCAG	NM_017957
	rev	TCAGGCTCTCTGTCCCGCTGATG	
ID1	fwd	AACGGCGAGATCAGCGCCCT	NM_002165
	rev	TGTTCTCCCTCAGATCCGGCGA	
BMP7	fwd	TCACAGCCACCAGCAACCACTG	NM_001719
	rev	ACCATGAAGGGCTGCTTGTTCT	
ID2	fwd	GCAGATCGCCCTGGACTCGCA	NM_002166
	rev	CACCGCTTATTCAGCCACACAGTGC	
LOX	fwd	TTACCCAGCCGACCAAGATATTCCTG	NM_002317
	rev	TTGTGGCCTTCAGCCACTCTCCTC	
TGM5	fwd	AGGCTGGCAGGTGCTGGACG	NM_004245
	rev	CCTGGACGAGCCAGGACATGC	
DUSP14	fwd	CAGCGCACACTGGACTCTTGAGG	NM_007026
	rev	GTCCTTGGTAGCGTGCTGTGACCT	
GAPDH	fwd	GGATATTGTTGCCATCAATGACCC	NM_002046
	rev	TCTCGCTCCTGGAAGATGGTGA	

## Supplementary Materials

The supplementary materials are available online at http://www.mdpi.com/1422-0067/19/5/1415/s1.

## Abbreviations

α-SMA	Alpha smooth muscle actin
ATP1β1	ATPase, Na^+^/K^+^ transporting, beta 1
BMP7	Bone morphogenetic protein 7
CRYAB	Crystallin alpha B
DSG4	Desmoglein 4
DUSP14	Dual specificity phosphatase 14
EGF	Epidermal growth factor
EGFR	Epidermal growth factor receptor
ERM	Ezrin/radixin/moesin
EPN3	Epsin 3
EMT	Epithelial-to-mesenchymal transition
ERK	Extracellular signal-regulated kinase
GEP	Gene expression profiling
hTCEpi	Telomerase-immortalized human corneal epithelial cell line
ID	Inhibitor of DNA-binding
LOX	Lysyl oxidase
MAPK	Mitogen-activated protein kinase
NGF	Nerve growth factor
SFs	Corneal stromal fibroblasts
SP	Substance P
TGF-β	Transforming growth factor beta
TGM5	transglutaminase 5
TNF	Tumor necrosis factor
ZEB1	Zinc finger E-box-binding homeobox1
