# Supplementary material for: Role of Bone Morphogenetic Protein 7 (BMP7) in the Modulation of Corneal Stromal and Epithelial Cell Functions
Source: Int J Mol Sci. 2018 May 9;19(5):1415. doi: 10.3390/ijms19051415 (PMC5983782; doi:10.3390/ijms19051415)
Supplement: Supplementary file 1 [file ijms-19-01415-s001.pdf]

Supplementary information:

Suppl Figure 1.

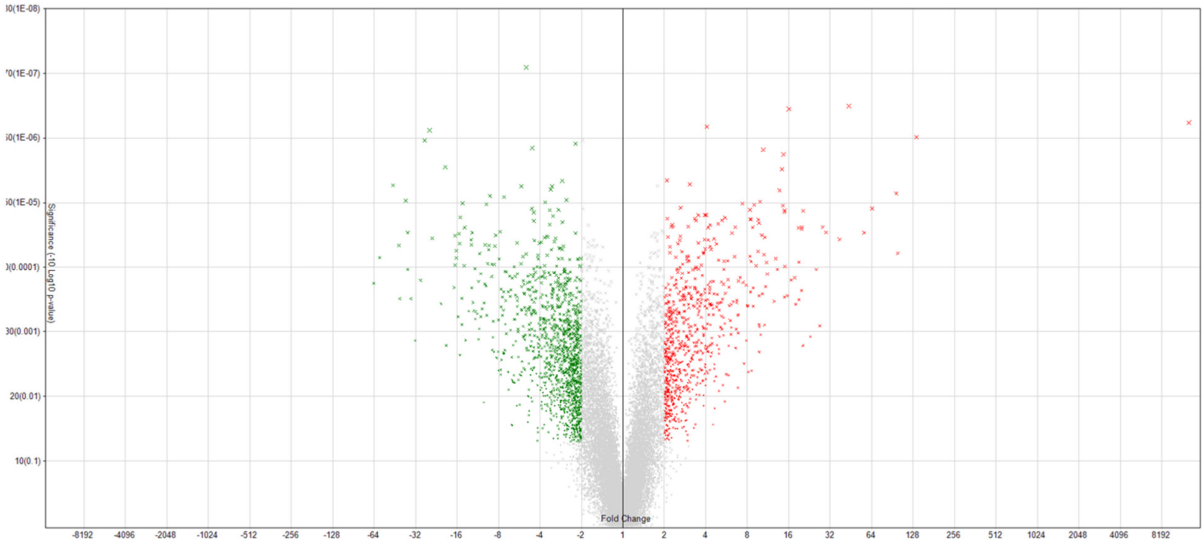

Suppl Figure 1. Volcano plot of the differentially expressed genes. X-axis shows the meseared fold changes in the expression whereas the y-axis shows the significance of the change in terms of negative log (base 10) of the *p*-value where more significant genes are plotted higher on the y-axis. The threshold used to select the differentially expressed genes is 2 for expression change and 0.05 for significance. Red marks show the genes upregulated and green marks show genes that are downregulated in BMP7 vs control analysis. Plot was generated using Affymetrix® Transcription Analysis Console (TAC) software (v.3.1).

Suppl Figure 2.

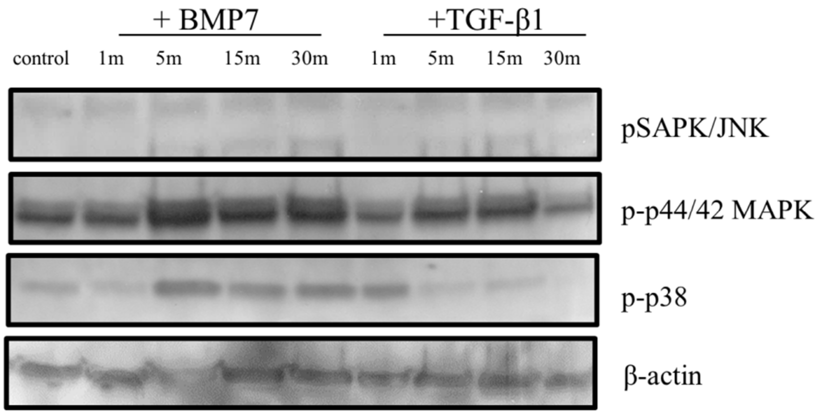

Suppl Figure 2. Activation of MAPK cascade proteins in hTCEpi cells upon stimulation with TGF-β1. Cells were stimulated with TGF-β1 (10 ng/ml) and cell lysates were collected at time points 1, 5, 15 and 30 minutes following stimulation. Phosphorylation of pSAPK/JNK, p44/42 MAPK and p-p38 proteins were analyzed with respective antibodies after immunoblotting. BMP7 stimulated corneal epithelial cell lysates were also collected at the same time-points for comparison.

Suppl Figure 3.

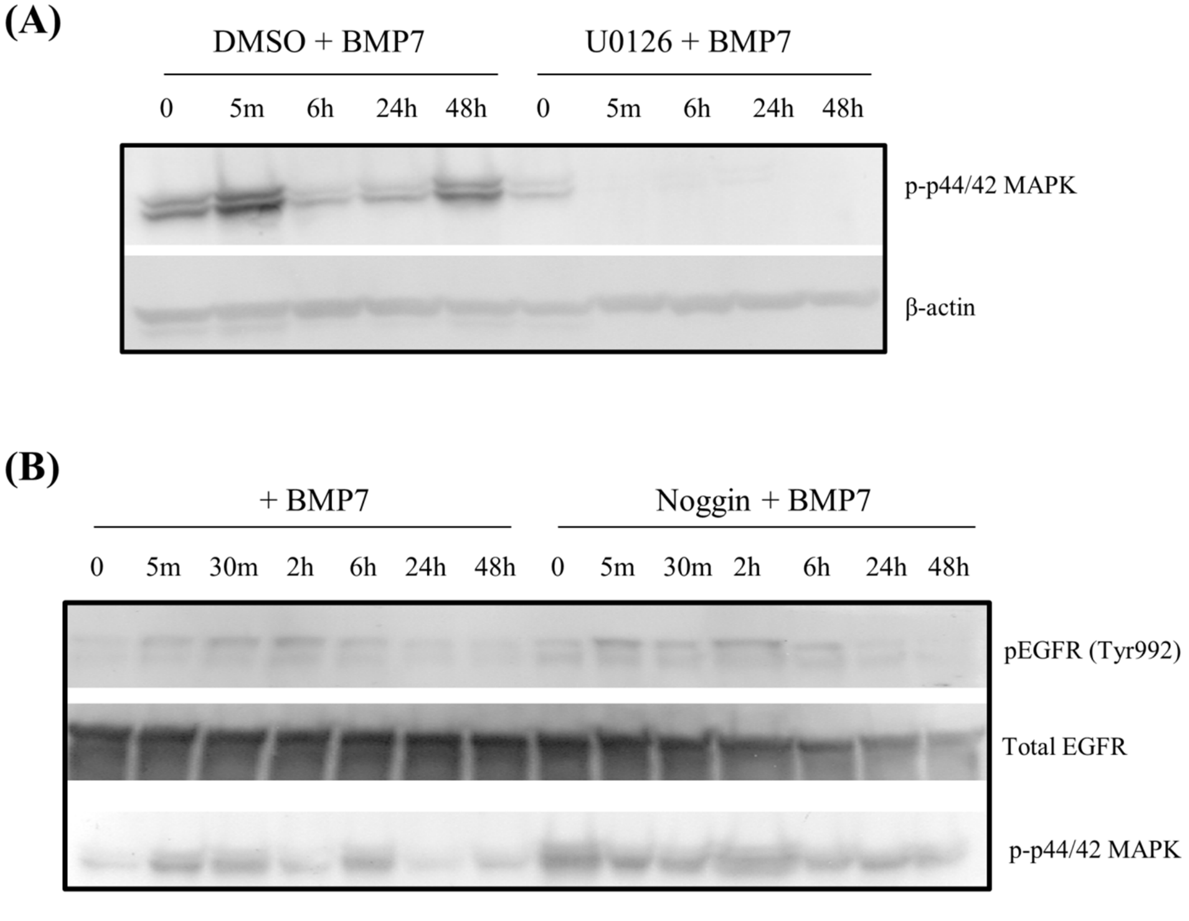

Suppl Figure 3. (A).Phosphorylation levels of p44/42 MAPK following BMP7 stimulation in the presence of MEK inhibitor U0126. Corneal epithelial cells were incubated with U0126 (10 μM) 30 minutes prior to BMP7 stimulation and cell lysates were collected at time points 5 min, 6, 24 and 48 hours and subjected to immunoblot analysis. Similarly, BMP7 treated cell lysates were also collected at the same time at different time points. Completely abolished phosphorylation of p44/42 MAPK was observed in the presence of U0126. (B). Phosphorylation levels of EGFR following BMP7 stimulation in the presence of its antagonist noggin. Corneal epithelial cells were incubated with rh noggin (100 ng/ml) 30 minutes prior to BMP7 stimulation and cell lysates were collected at time points 5 min, 30 min, 2, 6, 24 and 48 hours and subjected to immunoblot analysis. Similarly, BMP7 treated cell lysates were also collected at the same time at different time points. An increase in the phosphorylation of p44/42 MAPK was observed without any significant difference in the phosphorylation of EGFR at Tyr992.

Suppl Figure 4.

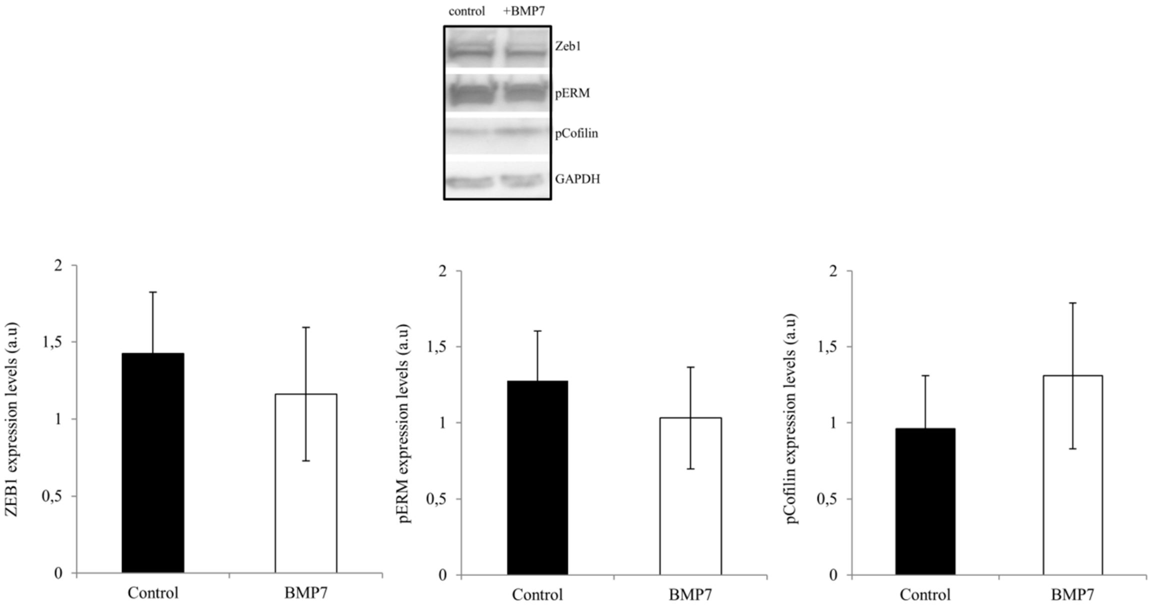

Suppl Figure 4. primary human CFs were stimulated with BMP7 for 24 hours and collected cell lysates were subjected to immunoblot analysis. An increase in the phosphorylation of cofilin and a decrease in the phosphorylation of ERM and Zeb1 were observed following BMP7 stimulation. Data represent the mean of the protein expression levels (± SEM of 3 independent experiments) shown as arbitrary units.

Suppl Figure 5.

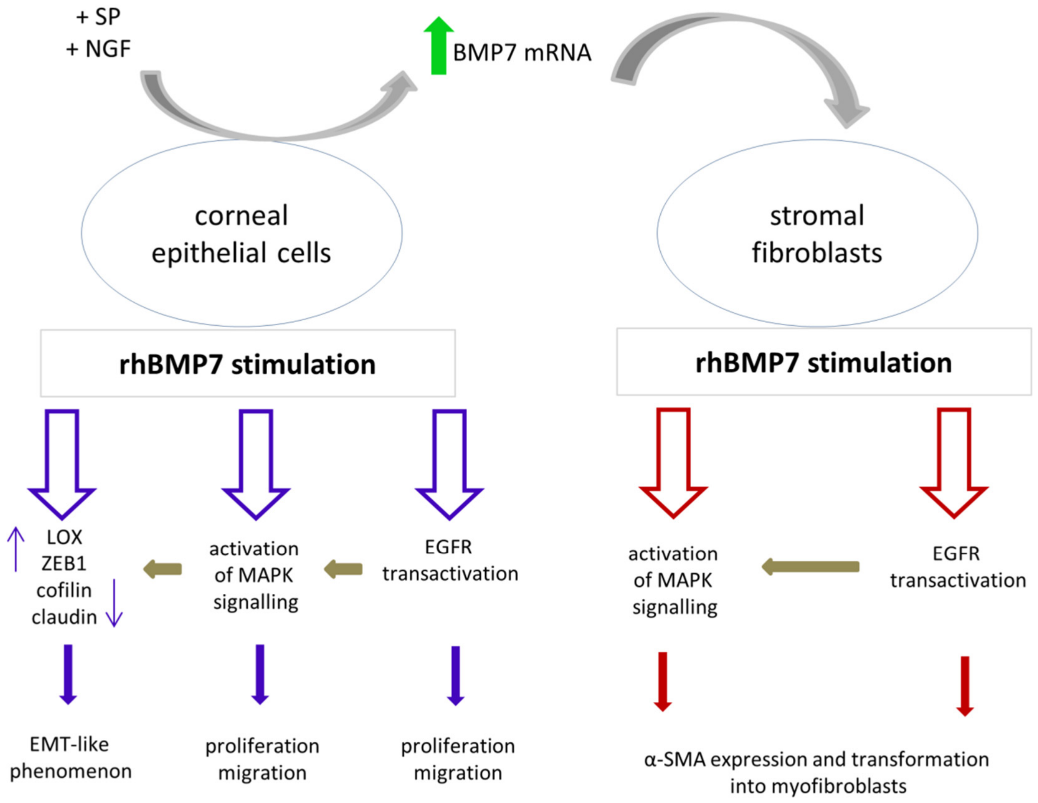

Suppl Figure 5. Stimulation of corneal epithelial cells with SP and NGF resulted to the increased expression of BMP7 mRNA. Corneal epithelial cell induced BMP7 can also have its influence on the function of stromal fibroblasts (SFs). Stimulation of corneal epithelial cells with exogenous rhBMP7 resulted to the activation of MAPK signaling. Furthermore, BMP7 stimulated EGFR transactivation also activates MAPK cascade. Simultaneously activated MAPK proteins can enhance EMT-like response in epithelial cells. The observed increase in LOX and ZEB1 expressions along with decreased cofilin and claudin expressions, in turn, mediate key roles during EMT-like transition of epithelial cells. All these events eventually lead to cell proliferation and migration necessary for wound healing. Similarly, BMP7 induced activation of MAPK proteins and EGFR transactivation in SFs also have influence in enhancing the a-SMA expression and transformation into myofibroblasts.
